# Survey Says: “COVID-19 Lockdown Hits Young Faculty and Clinical Trials”

**DOI:** 10.1016/j.stemcr.2020.06.010

**Published:** 2020-06-22

**Authors:** David G. Kent, David J.H.F. Knapp, Nagarajan Kannan

**Affiliations:** 1 York Biomedical Research Institute, Department of Biology, University of York, York, UK; 2 Institut de recherche en immunologie et en cancérologie, and Département de pathologie et biologie cellulaire, Université de Montréal, Montreal, QC, Canada; 3 Division of Experimental Pathology, Department of Laboratory Medicine and Pathology, Center for Regenerative Medicine, and May Clinic Cancer Center, Mayo Clinic, Rochester, MN, USA

## Abstract

COVID-19 has severely impacted laboratory research. Analysis of the International Society for Stem Cell Research (ISSCR) member survey has highlighted a particular impact on clinical trials and early-career investigators. The stem cell community needs to support young researchers and ensure that stem cell medicine does not lose its momentum.

## Main Text

These days, it is nearly impossible to avoid discussions on the current pandemic (WHO, 2020) and its impact on human lives, global health, science, and society at large. Organizations and businesses across the world have grappled with the practicalities of lockdown and there is a steady stream of novel approaches to working from home, desperately trying to minimize the damage to their long-term success. In such times, it is easy to get caught up with the most extreme anecdotes and equally easy to lose sight of the broad trends in how COVID-19 is impacting individuals. We were therefore excited to see that the ISSCR took it upon themselves to survey stem cell researchers to assess the global picture of how lockdown is impacting individuals and how we might sensibly plan ahead to mitigate the long-term damage to our field. The survey was conducted from 6 to 15 April, 2020 and filled out by 762 researchers from 52 countries, and it captured opinions from across all career stages. The data accompanied by a high-level summary are posted online (ISSCR, 2020a) and the ISSCR kindly granted us access to an anonymized version of the complete survey data, which we felt had some deeply troubling patterns, including a severe impact on early-career faculty and stem cell clinical trials. The opinions expressed in the article below represent the perspectives of the authors, who are early-career investigators and have been informed by several eminent colleagues in North America, Europe, and Australasia reached through personal communications.

We sought to dig beyond the general trends and shed some light on areas specific to stem cell research and stem cell researchers. We identified key areas that have been negatively impacted, including precious cell lines, mouse work, and clinical trials. We emerged with deep concerns for those on, or about to go on, the job market; those in the early stages of building their independent laboratories; and those with significant caring responsibilities. Among this turmoil, however, there was also a bevy of ideas and potential opportunities for stem cell research during and following the lockdown period, and we hope that as a community we can focus on building solutions in a broad and meaningful way that will ensure that stem cell research emerges from this crisis on strong footing.

### Interrupted Clinical Trials: A Silent Sub-epidemic?

**Figure 1:**
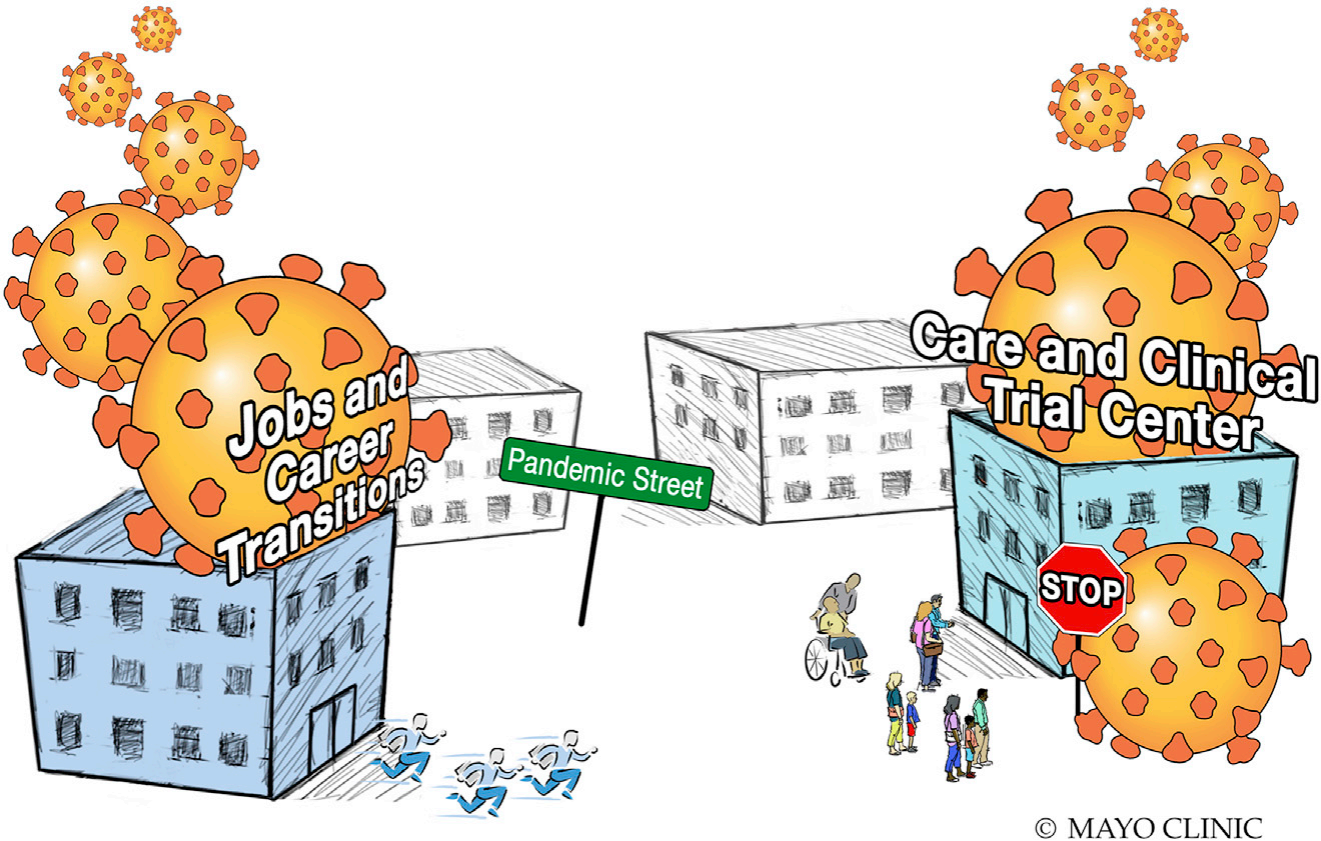
Covid-19 Hits Research Careers and Clinical Trials An illustration depicting state of the young investigators and clinical trials during the Covid-19 lockdown.

Lockdown has halted numerous clinical trials around the world (Lupkin, 2020). Many of these trials provide access to medical options for patients where standard-of-care does not exist. Nearly 13% of survey participants were involved in clinical trials and >80% of these were negatively affected by COVID-19. This suggests an early disturbing sign that may only be the tip of the iceberg. While hospitals have remained open as essential services during the lockdown, most elective procedures have been postponed, if not suspended (Frankel and Romm, 2020) (Figure 1). Hospitals across countries have transformed into COVID-19 facilities, prioritizing COVID-19-related patient care and trials. Patients needing care at hospitals do not have access unless it is critical, and those who need critical care or are on trials requiring follow-up may fear going to hospitals due to safety concerns (Bernstein and Sellers, 2020). The Mayo Clinic is a case in point as one of the largest clinical trial centers in the USA that now finds itself under extreme financial strain due to the current COVID-19 crisis. The impact in the Mayo Clinic Center for Regenerative Medicine where stem cell trials are regularly conducted includes operational budget reductions as well as temporary furloughs and reduction in FTE levels for staff. This has negatively impacted their ability to sustain the work ongoing for many projects, including 20 regenerative medicine clinical trials. We suspect the situation could be much worse in other clinical trial centers with fewer resources, and while we assume that the COVID-19 disruption is temporary, there are serious threats for some of these trials to be significantly delayed at best and completely abandoned at worst.

### Research Not Immune to COVID-19 Impact

As expected, and as echoed across research from a number of other organizations, the COVID-19 lockdown has had a dramatic impact on scientific research. The survey revealed that nearly 85% of research laboratories and their activities were impacted by COVID-19. Some laboratories have switched to COVID-19 research while 65% of laboratories were mostly or completely shut (ISSCR, 2020a). Numerous graduate students in the middle or near the end of their training have had substantial delays imposed with over 70% “certain” or “unsure” about delays in defense/graduation date. In most countries, the lockdown has lasted more than 3 months (at the time of writing). Many laboratories had to curtail or completely abandon long-term experiments and the initial uncertainty and need to rapidly reduce laboratory activity has particularly impacted animal experiments and lengthy cell culture experiments. Researchers on average estimated that getting laboratory research back at a reasonable level will take half a year and, in some cases, up to 2 years (including key tasks such as reviving cell lines from cryopreservation, expanding animal colonies, etc.). The path to return their research activities to pre-COVID-19 status would greatly depend on how long lockdown lasts. There will be permanent losses in this process as well, particularly due to a rapid shutdown of lab work that could not be done comprehensively. Like many areas of laboratory research, this pandemic has dealt a severe blow to productivity in stem cell biology and medicine.

There are many other ways that research has been impacted. Job interviews have been cancelled and hiring processes have been frozen, and widespread furloughing has created uncertainty and anxiety due to possible job losses if the situation gets worse. Cross-border travel has been virtually halted and a number of reagent supply lines have been disrupted, including access to good-quality personal protective equipment. Rapidly evolving policies have added more confusion and stress to investigators and institutions trying to decide when it is safe to re-initiate research. On a scale of stress of 0 (no stress) to 100 (maximum stress), survey respondents scored an average of 64 with approximately one-third of respondents scoring very high (80+). We have also seen nearly all scientific meetings being cancelled, postponed, or switched to a virtual meeting format, including the ISSCR holding its first virtual annual meeting this year (ISSCR, 2020b).

That said, the stem cell community has already begun to demonstrate its resilience. More than 20% of researchers have shifted their work to try and tackle aspects of COVID-19 biology. Others have initiated new global communities to interact despite the lockdown, inspiring new research and new collaborations for when the shackles are removed. Perhaps the largest piece of good fortune for science is the amount of data analysis that has occurred in the past weeks with many hands and minds having been liberated from cell culture hoods and molecular experiments.

When asked about the most important lesson from this crisis, a veteran researcher, Dr. Connie Eaves, replied, “The general respect and confidence [afforded to]… all sectors in science and scientists to provide the most reliable guidance on how to navigate through this global ‘Covid experience’ is a reassuring tribute to the esteem the research community has earned through its past performance and now demonstrated [via its] immediate rise to new challenges with creativity and insistence on reliable data.”

### The Biggest Impact on Stem Cell Scientists: Precarious Career Stages and Caring Responsibilities

When the world shuts down and institutions assess their prospective financial position, it is uncommon to see major hiring campaigns. This places those on, or nearing, the job market in a particularly precarious position (even more so than before COVID-19!) and the signs of stress were clearly evident in the survey responses. Indeed, when asked to rank level of concern from 1 to 5 with 5 being highest across a range of questions (including project timelines, current and future manuscripts, and current and future grants and career advancement), mean concern across all questions was 3.7/5, with the effect on research timelines being the area of greatest concern (4.3). One respondent summed it up well: “Unfortunately, the main thing I am losing is time and money.” Many of us are feeling this pressure and research funders and universities and hospitals (some more so than others) have devised plans to mitigate damages to currently funded research. Still, it is obvious from the survey that such assurances are not placating the fears of those in very unstable positions, and these (mostly young) scientists need our support as a community.

To make matters worse, many of the grant competitions we rely on have been postponed or cancelled as a result of the pandemic, with uncertain prospects for some organizations if they will return at all. This, coupled with the build-up of applications in subsequent cycles, the redirection of funds to COVID-19 research, and the shadow of a global economic depression looming (WTO, 2020) make many of us worried for the future of our labs. While in some cases bridge funding has been made available to cover the cancelled competitions, this is by no means universal, and does little to help early-career researchers who have not yet obtained an external grant to bridge. Further, in many cases critical experiments for nearly finished papers languish uncompleted while contract end dates and grant reporting deadlines loom closer and closer.

Caring responsibilities (predominantly children, based on free-form responses) was the other area that was hitting individual scientists quite hard. A particularly illuminating point from the survey came when breaking down the data by career stage. Two in three early career (0–8 years) faculty members cited caring responsibilities compared to 40% in the more senior (15+ years) faculty. While ages of children were not recorded, it stands to reason that small children are more common for early-career faculty members, meaning that a two-parent, two-child household almost certainly has to resort to shift work (and getting 2 × 40+ hour work-weeks accomplished becomes virtually impossible). The survey data supported this notion with 71% of early-career faculty reporting increased time commitment for caring and only 55% of more senior faculty. Somewhat depressingly, alternating work and childcare shifts was the sole suggestion for allowing uninterrupted work time when having young children at home.

This extra burden will potentially fly under the radar and go unaddressed when it comes to how we address the impact of COVID-19 on the careers of early-career researchers. Moreover, it is more likely that this extra burden will fall on female researchers, leading some to suggest that COVID-19 should be treated like a period of parental leave when it comes to grant funding and career advancement (Minello, 2020). One respondent was particularly frustrated with senior leaders and their lack of empathy with such circumstances: “How many people are still getting the message from their leadership that they need to be maximally productive right now by writing grants, papers, etc.? […] All the attempts to remind leadership that many of us (especially junior people) are home with small children have no effect. Are they not hearing it or do they really not care?” Since the survey, hundreds of universities have proactively extended the promotion/tenure clock for junior faculty members by 1 year due to COVID-19 (Weissman, 2020), but this will only go so far to address this singular concern and does not address many other aspects of how lockdown has affected different groups of people in different ways.

It is clear that we need to sit down as a stem cell community and determine how to best support our young researchers who find themselves disproportionately impacted in a negative way, and we hope that senior leaders might consider ways to alleviate this additional strain, both financially and in a career-focused manner.

### The Silver Lining: Creative Approaches and Novel Opportunities

With many of us banished from the lab, we have been forced to adapt operations in many ways. To continue minimal operations safely, survey respondents shared many useful tips including splitting work into multiple shifts and online booking systems for all work areas and instruments to ensure that minimal numbers of people are physically present at any given time. Another interesting adaptation was the use of shared electronic lab notebooks to allow multiple individuals to perform routine maintenance on ongoing long-term experiments. Such adaptations allow physical distancing and also provide a valuable blueprint for how to ramp back up safely as restrictions are loosened.

While adaptations to allow essential work to proceed are indeed important, this still leaves some scientists with a surplus of time usually filled with lab experiments. Again, survey respondents came forward with suggestions on how to stay productive while at home, which can be particularly challenging in the face of larger life issues and/or caring responsibilities. Two of the most common tasks, perhaps unsurprisingly, were to analyze data and write manuscripts. This is great if your project or projects are at this stage, but for many, new questions are being formulated through the discovery of publicly available high-content datasets through the many open access repositories (Sequence Read Archive [SRA], Gene Expression Omnibus [GEO], and FlowRepository to name just a few). While it may not be the perfect experiment in your eyes, there is often something to find, and you certainly can’t argue with the price!

Another activity that is being taken up in droves by investigators of all stages is the planning of new projects and grant applications. While the inability to generate preliminary data is certainly a drawback, having a grant pre-written and experiments pre-planned certainly won’t hurt for subsequent competitions. As such, many respondents reported the pursuit of new skills with the most popular focus being improving computational and bioinformatic skills.

Working from home itself has its drawbacks too. One useful suggestion was simply to set a schedule and stick to it. This, combined with setting aside a dedicated space for work that is free of distractions, can help keep focus through those long undifferentiated days. Social contact through a variety of team meetings has also been implemented by many using platforms including Zoom, Skype, Slack, and many others. Short informal meetings with frequencies up to daily in addition to regular group meetings have helped lab members stay connected according to many reports. Further, these allow some external motivation through accountability and thus can help with productivity. This can, however, be a curse as well as a blessing depending on individual circumstances.

Beyond lab meetings, job interviews, seminars, and even full conferences are now being conducted on these virtual meeting platforms. A number of individuals have used this period of physical isolation to reach out to new collaborators and the scientifically interested general public over social media. Connecting to our colleagues is more important now than ever as isolation can quickly take a major toll on our mental health otherwise.

It goes without saying that many practical, productive, and creative solutions have been implemented by members of the ISSCR community. There remain, however, a number of unresolved questions that survey respondents posed that deserve consideration. For example, how might stem cells be applied to understanding COVID-19 and to improving treatment options (ISSCR, 2020a)? Many highly trained stem cell biologists were attracted to the field by a desire to help improve human health, so many of us are now asking the question of how we can contribute. To this end, the ISSCR has begun to host weekly calls to facilitate discussion on how best to continue all of our important work and contribute to the fight against COVID-19 (ISSCR, 2020a). There are inevitably many inventive grant applications en route that will look into such treatments, and many respondents reported working in this area already: developing testing methods, creating organoid model systems, contributing to vaccine development and drug screening, testing immunomodulation strategies, and providing logistics support for virology groups and first responders. This is, of course, stimulated in large part by the wave of COVID-19 funding that is becoming available. While this money is indeed an opportunity, we must, as a community, make absolutely certain that our proposals are scientifically sound in this area where most of us are not fully expert, a sentiment echoed by many respondents. This is not a “cash grab” opportunity.

Another area for debate came from authors and reviewers: how should lab closures affect how you peer review, and how should you answer reviewer comments when experiments cannot be performed? It was heartening to see *Stem Cell Reports* implementing several measures for our community members struggling to publish their work during this crisis (Pera, 2020). Sharing his perspective on the matter, Dr. Martin Pera, *Stem Cell Reports* Editor-in-Chief, said, “I am old enough to remember when a *Nature* paper had four figures and no supplemental material. People managed to publish good findings in that format from time to time. I would like to see some resurgence of short, cogent reports of new significant findings.” Perhaps as a community we should consider taking this opportunity to stop demanding additional experiments where they may not be absolutely necessary. Instead, we could simply allow authors to plainly describe the limitations and uncertainties that remain based on the available data rather than maintain the illusion of perfection that many journals have now come to expect.

### Conclusion

From the crippling of ongoing and planned clinical trials across the full breadth of stem cell research to the devastating loss of productivity for those researchers near career transitions, the ISSCR survey has revealed some of the bleak truths on the impact of COVID-19 in the stem cell community. This pandemic has proven a challenge that without the right mitigations may prove insurmountable for many. It falls to us as a community to band together to make sure that no budding researchers fall through the cracks. As with combatting the virus itself, this comes down to continued monitoring of the situation. This together with follow-ups to assess the ultimate impact of the pandemic on career paths, clinical trials, and adaptation may help efforts to mitigate the effects of this and future pandemics. Amidst such challenging times, it is inspirational to see members of the community adapting in creative ways to keep advancing stem cell research and applying it to the fight against COVID-19. This spirit of adaptation and collaboration is needed now more than ever as we establish the new normal under which we will have to operate in the coming years.
